# Fathers Matter: Enhancing Healthcare Experiences Among Fathers of Children With Developmental Disabilities

**DOI:** 10.3389/fresc.2021.709262

**Published:** 2021-07-21

**Authors:** Tatiana Ogourtsova, Maureen E. O'Donnell, Derrick Chung, Frank Gavin, Aline Bogossian, Annette Majnemer

**Affiliations:** ^1^Research Center of the Jewish Rehabilitation Hospital, Centre intégré de santé et de services sociaux de Laval, Site of Centre for Interdisciplinary Research in Rehabilitation of Greater Montreal, Laval, QC, Canada; ^2^Centre for Interdisciplinary Research in Rehabilitation of Greater Montreal, Montreal, QC, Canada; ^3^School of Physical and Occupational Therapy, Faculty of Medicine and Health Sciences, McGill University, Montreal, QC, Canada; ^4^Provincial Health Services Authority, Vancouver, BC, Canada; ^5^Department of Pediatrics, Faculty of Medicine, University of British Columbia, Vancouver, BC, Canada; ^6^The Research Institute of the McGill University Health Center, Montreal, QC, Canada; ^7^School of Social Work, University of Montreal, Montreal, QC, Canada; ^8^Montreal Children's Hospital, Montreal, QC, Canada

**Keywords:** health-care experiences, barriers and facilitators, interactions with health-care professionals, clinical practice, family-centered approach, father, children with disabilities

## Abstract

**Background:** Being a parent of a child with a developmental disability (DD; e. g., cerebral palsy, autism) comes with great challenges and apprehensions. Mothers and fathers of children with DD are experiencing heightened levels of psychological distress, physical health problems, financial difficulties, social isolation, and struggles with respect to traditional parenting roles. In relation to the latter, the involvement of fathers in caregiving in today's society is increasing and is highlighted by its importance and positive contribution to the development of their children. However, fathers of children with DD report feeling excluded and marginalized by healthcare providers (HCPs) when arranging for and getting involved in healthcare services for their children. Currently, there is limited evidence as to what factors influence those experiences. We aimed to explore barriers to and facilitators of positive and empowering healthcare experiences, from the perspectives of fathers of children with DD and HCPs.

**Methods:** A mixed-method approach, such as quantitative (survey) and qualitative (semi-structured interview) strategies, was used. Participants were fathers of children with DD and HCPs working in childhood disability. Data analysis consisted of using descriptive statistics and an inductive-thematic analysis of emergent themes.

**Results:** Fathers (*n* = 7) and HCPs (*n* = 13, 6 disciplines) participated. The fathers indicated that while they were moderate to very much satisfied with their interactions with HCPs, they reported that HCPs were only sometimes attentive to them during interactions. Fathers also revealed that positive interactions with HCPs in relation to their children had multiple benefits. Several themes related to barriers and facilitators of optimal interactions and parent–professional relationships emerged. These included session factors (time, attention), personal factors (knowledge of the condition, child and healthcare system, acceptance vs. denial, previous experiences, culture, stereotypes, pre-existing beliefs, stress levels, working schedule), and family dynamics. The participants offered several insights into the different strategies that can be implemented to promote optimal interactions between fathers and HCPs.

**Conclusion:** We identified several barriers, facilitators, and improvement strategies for optimal interactions and enhanced parent–professional relationships from the perspectives of fathers and HCPs. These can be integrated by existing clinical settings in efforts to enhance current clinical practices and improve child- and parent-related outcomes.

## Introduction

Becoming a parent, in itself, is a stressful life event. Becoming a parent of a child with a disability imposes even greater challenges and concerns, as parents now need to adjust to provide care for a child with emergent and frequently changing healthcare needs [reviewed in (1)]. Both mothers and fathers of children with disabilities are reported to experience heightened levels of stress and psychological distress (2, 3), physical health problems, financial difficulties, social isolation, depression, and conflict with traditional gender roles (4, 5). Moreover, for the last five decades, the amount of time fathers spend with their children has increased dramatically (6). Currently, fathers are acquiring a wide range of roles and responsibilities beyond the traditional ones, where they are becoming more conscious and aware of being models of social and emotional behavior for their children (7). In fact, the involvement of the father in childcare is reported to result in positive socio-emotional, cognitive, and developmental outcomes of their child [reviewed in (8)], as well as in improvements to the emotional well-being and stress levels of the mother (9).

However, despite the increasing involvement of fathers in caregiving, as well as its important and positive contribution to child development, research in the field of childhood-onset disability is still primarily focused on mothers (10–12). For instance, while the applications of family-centered approaches involving parents are on the rise, these are mainly directed toward and largely used by mothers (13). The need to consider fathers, however, and the challenges this can present are increasingly recognized (10). For example, preliminary evidence suggests that fathers of children with disabilities feel excluded and marginalized by healthcare providers (HCPs) when arranging for and getting involved in healthcare services provided for their children (14).

Communication of parents with healthcare professionals in relation to their child with disability reveals a great deal. For example, a pilot study examining a coping intervention for parents of children with disabilities determined that the amount and the quality of the communication with HCPs were a primary predictor of how fathers managed condition-related stress levels (15). Similarly, the quality of parent–professional relationships was hypothesized to influence these interactions (1) and ensure the understanding of the father about the condition of their child (16). Previous efforts have been made to describe the healthcare experiences of fathers of children with (10) and without disabilities (8). Nonetheless, to the best knowledge of the authors, there is limited evidence as to what factors influence the father-healthcare professional interactions, both from the points of view of fathers and healthcare professionals.

With the aim to promote beneficial and helpful interactions and experiences for fathers of children with developmental disabilities, the goal was to explore the barriers to and facilitators of positive interactions and empowering parent-healthcare professional relationships, from the perspectives of fathers of children with developmental disabilities and healthcare professionals (HCPs).

Where,

…*children with developmental disabilities* are children (0–18 years old) with primary motor impairments (e.g., cerebral palsy, muscular dystrophies, spina bifida, spinal muscular atrophies, etc.) and/or with developmental behavioral conditions (e.g., autism spectrum disorders).

…*father* is the male parent in relation to the child (biological, adopted, foster, step).

…*healthcare service experiences* include interactions with HCPs for diagnostic, treatment or medical follow-up purposes related to the disability of the child, in different healthcare settings (e.g., hospitals, rehabilitation centers, community clinics).

…*HCPs* include all-specialty physicians and surgeons, nurses, occupational and physical therapists, speech language pathologists, social workers, special educators, etc.

## Materials and Methods

### Study Design

A mixed-method study design was used, including quantitative (Likert-scale survey) and qualitative (semi-structured interview) methods. In addition, we adopted a patient-oriented research methodology. This was achieved by recruiting two parent advisors to be part of the research team prior to protocol development (DC & FG). They are both fathers of children with disabilities. The parent advisors were involved in the following activities in relation to the project: development of study protocol, development of measurement tools (survey and structure of the interview), data interpretation, and review of ensuing knowledge translation material (presentations, present manuscript).

### Study Population

Populations of interest included HCPs and fathers of children with DD. The inclusion criterion for HCPs was to be a licensed HCP, working in the field of childhood-onset developmental disability for ≥6 months, and providing direct assessment and/or intervention for a minimum of 50% of their working time. The exclusion criterion was (1) research or medical assistant personnel (e.g., administrative assistants) who are not directly involved in procedures related to diagnostics and/or treatment and follow-up of children with DD. For fathers of children with DD, the inclusion criteria were (1) male gender and (2) being a father (biological, adopted, step, foster) for ≥6 months of a child with a diagnosed DD (e.g., cerebral palsy). All the participants needed to be fluent in English or in French.

### Sample Size Consideration

We aimed to recruit a sufficient number of participants to achieve data saturation. An effort was made to recruit HCPs from different disciplines and fathers of children with different disabilities and various age groups.

### Source of Data

The participants were recruited using word-of-mouth/snowball/convenience sampling techniques at the collaborative healthcare services points. These included the Montreal Children's Hospital, Shriners Hospital for Children (Montreal, Quebec, Canada), and the BC Children's Hospital, Sunny Hill Health Centre for Children (Vancouver, BC, Canada). Recruitment was performed via wall-mounted/email/web-posted advertisements. Ethical approval was obtained from all participating clinical sites and informed consent was provided by all the recruited participants prior to their engagement with the study.

### Study Procedures

First, the participants were asked to fill out a demographic form and a short Likert-scale based survey that was developed in-house and reviewed by all team members, including patient advisors (Supplementary Material 1). For the father-participants, the survey aimed to gather information about their involvement in the healthcare of their child and overall perceptions regarding their experiences and interactions with HCPs in the past. For the HCPs, the survey was designed to gather their perspectives about the involvement of fathers in the healthcare of children and their experiences and interactions with fathers in their clinical practice. Individual interviews were then conducted online using Zoom (Zoom Video Communications, Inc., San Jose, CA, United States). The interviews followed a semi-structured format that was co-developed by the team, including patient-partners (Supplementary Material 1). The interviews included introductory statements, open-ended theme-related questions, and interview terminations using closing remarks and a summary of discussed topics/points raised. The interviews ended when no new ideas emerged following the summary/closing remarks statement (i.e., data saturation was reached).

### Variables Collected

Demographic variables for HCPs included their discipline and degree, practice clinical setting, amount of experience in the field of childhood disability, age, gender, race/ethnicity, population served, and time spent on continuing education. Demographic variables for fathers of children with disabilities included their age, race/ethnicity, relation to the child (biological, foster, stepparent, adopted), marital status, education level, employment status, and information about the child with a disability (age, condition, rank in the family).

Main variables collected (from the perspectives of both study groups) included (1) the perception of involvement of fathers in healthcare of their child; (2) satisfaction and comfort level in interactions between HCP and fathers of children with disabilities; (3) influential factors (barriers and facilitators) impacting experiences/interactions of fathers of children with disabilities with HCPs; (4) effects of involvement of fathers (from the perspective of HCPs only) and effects of interactions with HCPs (from the perspective of fathers of children with disabilities only) on personal, child-related, and family-related outcomes; and (5) strategies and recommendations to enhance interactions and parent–professional relationships and promote family-centered care.

### Data Management and Analysis

Descriptive statistics were used to summarize demographic data. Audio data from the interviews were transcribed verbatim. The NVivo® software (QSR International, Doncaster Australia) was used for data management. Triangulation methods were used for data analysis (17). More specifically, one author (TO) read all the transcripts to gain a general sense of the meaning of the content. The content of the transcripts was then analyzed by generating initial codes for all meaningful ideas emerging from the data using a directed content-based analysis technique (18). Following this, a second coder (research assistant who was not a study participant and did not assist with the interviews in any way) verified the coding grid. Discrepancies were discussed with both raters to explore their meaning and/or relationship to other codes, and a consensus was reached. A final round of analysis was then performed by the first coder to ensure that all relevant statements were coded and that agreement between raters was 100% for all statements.

## Results

### Demographics of Participants

Seven (*n* = 7) fathers of children with disabilities were recruited. The father-participants were 42.6 ± 8.2 years old, 100% Caucasian, all biological parents to their children, all fulltime workers, four (*n* = 4) were married, and three (*n* = 3) were single. Their education level ranged from a college professional diploma (*n* = 3), Bachelor (*n* = 3) to Masters (*n* = 1). Their children with disability (male: *n* = 4; female: *n* = 3) were on average 9.4 ± 5.3 years old (range: 18 months−15 years) with the following conditions: epilepsy, anxiety, cognitive and language delay, trisomy 21, cerebral palsy, autism spectrum disorder, attention deficit and hyperactivity disorder, developmental coordination disorder, behavioral challenges, and globoid cell leukodystrophy.

Thirteen (*n* = 13) HCPs were recruited. HCPs (female: *n* = 10; male: *n* = 3) were 37.8 ± 13 years old. Degrees obtained included Bachelor (*n* = 1), Masters (*n* = 8), Medical doctor (*n* = 1), and PhD (*n* = 2), with diplomas obtained anywhere from 1982 to 2017. The HCPs were from the following disciplines: Occupational Therapy (*n* = 6) Speech Language Pathology (*n* = 2); Neuropsychology (*n* = 2), Pediatrics (Medical Doctor, *n* = 1), Social Work (*n* = 1), and Nursing (*n* = 1). Most of them were working fulltime (*n* = 10) in the field of childhood disability for an average of 11.5 ± 9.7 years. Fields of practice included general neurodevelopment and autism spectrum disorder (*n* = 7), orthopedics (*n* = 2), neurotrauma (*n* = 2), intellectual disability (*n* = 1), and complex care (*n* = 1). Clinical settings included university teaching hospitals (*n* = 5), rehabilitation centers (*n* = 3), community or private clinics (*n* = 3); and a mix of clinical settings (*n* = 2). On average, the recruited HCPs spent 10.2 ± 12.3 h per month on continuing education.

### Survey Responses: Involvement of Fathers in Healthcare and Satisfaction/Comfort Level of Child in Interactions With HCPs

Tables 1A,B outline response frequencies to the initial survey regarding the involvement of fathers in the healthcare of their child, and satisfaction/comfort level in interactions from the perspectives of both groups.

**Table 1A:** Response frequencies of father-participants: involvement in healthcare and satisfaction/comfort level of child in interactions with HCPs.

		**Not all all** **Never** ***n* (%)**	**Slightly** **Sometimes** ***n* (%)**	**Moderately** **Often** ***n* (%)**	**Very much** **All the time** ***n* (%)**
*I am involved in setting up/arranging/organizing health-care services for my child*.		2 (28.5)		2 (28.5)	3 (42.8)
*I am involved in taking my child to his/her health-care services appointment(s) and/or emergency visit(s)*.			2 (28.5)	2 (28.5)	3 (42.8)
*I am present (with my spouse/partner, if applicable) when my child is receiving health-care services*.			1 (14.2)	4 (57.1)	2 (28.5)
*I advocate for my child's health-services*.		1 (14.2)	2 (28.5)		4 (57.1)
*I am taking part in applying treatment recommendations that my child receives*				2 (28.5)	5 (71.4)
*Overall, I am satisfied with the interactions/communication I had with HCPs in the past*.			2 (28.5)	2 (28.5)	3 (42.8)
*HCPs are generally attentive to me*.			4 (57.1)	1 (14.2)	2 (28.5)
*I feel generally comfortable with the HCPs*.			1 (14.2)	1 (14.2)	5 (71.4)
*I feel generally understood by the HCPs*.			1 (14.2)	3 (42.8)	3 (42.8)
*I feel generally supported by HCPs*.			2 (28.5)	3 (42.8)	2 (28.5)
*I feel that generally I formed and continue to have good relationships with the HCPs*.				3 (42.8)	4 (57.1)
*I had positive/good experiences/helpful interactions with HCPs*.				1 (14.2)	6 (85.7)
*I had negative/bad experiences/not helpful interactions with HCPs*		4 (57.1)	1 (14.2)	2 (28.5)	
**Response frequency (%)**	**Color-code**				
0 to <25					
25 to <50					
50 to <75					
75–100					

**Table 1B:** Response frequencies of HCPs: satisfaction/comfort level in interactions with fathers and involvement of father in healthcare of child.

		**Not all all** **Never *n* (%)**	**Slightly** **Sometimes** ***n* (%)**	**Moderately** **Often** ***n* (%)**	**Very much** **All the time** ***n* (%)**
*Overall, I am satisfied with the interactions/communication I had with fathers of children with disabilities in the past*.			3 (23.0)	6 (46.1)	4 (30.7)
*Fathers of children with disabilities are generally attentive with me during interactions*.			3 (23.0)	7 (53.8)	3 (23.0)
*I feel comfortable interacting with fathers of children with disabilities*.			1 (7.6)	2 (15.3)	10 (76.9)
*I feel that fathers of children with disability understand me*.			3 (23.0)	5 (38.4)	5 (38.4)
*I feel that fathers of children with disabilities feel supported by me*.			4 (30.7)	4 (30.7)	5 (38.4)
*I feel that I form/continue a good relationship with fathers of children with disabilities*.			1 (7.6)	5 (38.4)	7 (53.8)
***The extent to which fathers***.					
…*advocate for their child*.			4 (30.7)	9 (69.2)	
…*implement and apply your recommendations/treatment regiments you are providing them with*.			6 (46.1)	7 (53.8)	
…*are involved in setting up/arranging/organizing health-care services for their child*.			9 (69.2)	3 (23.0)	1 (7.6)
…*are involved in taking their child to his/her health-care services appointment(s) or emergency visit(s)*.			6 (46.1)	7 (53.8)	
…*are present when their child is receiving health-care services (alone or with spouse/partner)*.			6 (46.1)	6 (46.1)	1 (7.6)
**Response frequency (%)**	**Color-code**				
0 to <25%					
25 to <50					
50 to <75					
75–100					

Father-participants reported that 57.1 to 85.7% of them were “Moderately (Often)” to “Very much (All the time)” involved in the healthcare of their child, such as organizing services, taking the child to their appointments and medical visits, being present during medical visits, and advocating for the healthcare of their child. All of them (100%) reported that, at all times, they are taking part in applying treatment recommendations that their child receives. In terms of their satisfaction and comfort level in interactions with HCPs, father-participants reported that 71.3 to 85.7% of them are “Moderately” to “Very much” satisfied with the interactions they have had with HCPs in the past, where they conveyed feeling comfortable, understood, and supported by HCPs. However, 57.1% reported that HCPs are only “Sometimes” attentive to them during interactions. All of them reported that they were able to form a good relationship with HCPs and had a positive/good experience/helpful interaction(s). Only 28.5% of them reported having frequent negative experiences in interacting with HCPs in relation to the care of their child.

On the other hand, 92.3 to 100% of the HCPs reported that fathers are only “Slightly (Sometimes)” to “Moderately (Often)” involved in advocating for the healthcare needs of their child, implementing and applying the recommendation/treatment regiments, getting involved in setting up healthcare services for their child, taking the child to medical visits, and being present during appointments. On average, 83.1% of the HCPs reported that they are satisfied with the interactions they have had with fathers of children with disabilities. HCPs reported that fathers are generally attentive to them and understand them, and that they (the HCPs) were comfortable in those interactions and were able to form good relationships. However, a lower percentage of the HCPs (69.2%) conveyed that fathers of children with a disability felt “Moderately” (30.7%) to “Very much” (38.4%) supported by them.

### Semi-structured Interviews

The initial agreement between the two independent coders of the semi-structured interviews was high at 96.6%. The few discrepancies in coding were resolved through discussion to reach a 100% consensus.

Four main themes emerged from the semi-structured interviews:

1. Impacts of the interactions between fathers of children with disabilities and HCPs (from the perspectives of the father-participants).
2. Impacts of fathers in the healthcare of their child with a disability (from the perspectives of the HCPs).
3. Barriers and facilitators to optimal and empowering interactions between fathers of children with disabilities and HCPs.
4. Solutions to optimize: (a) interactions between fathers of children with disabilities and HCPs and (b) involvement of fathers in the healthcare of their child.

Those four themes and their respective subthemes are described below, along with the most salient utterances from the study participants.

#### Impacts of Interactions (From the Perspectives of Father-Participants)

Father participants (FP) reported several impacts of positive (*n* = 6 subthemes, *n* = 14 utterances) and negative (*n* = 4 subthemes, *n* = 7 utterances) interactions with HCPs in relation to their child. Impacts of positive interactions included “Gaining a better perception and awareness of child's needs, challenges and strengths” (*n* = 7 utterances):

*FP3: “I think generally when I walk away from [positive interactions with HCPs], I am being reminded about [my child's] issues and that tends to have a positive effect generally on how I interact with [my child].”*

The participants also reported that positive interactions allowed them to “Focus on what is important” (*n* = 2 utterances) and “Bringing the family closer together” (*n* = 1 utterance):

*FP3: “Dealing with an issue with [my child] is something that is a scary trying thing on the family, and I think things like that tend to bring us closer together, and [my wife] and I always bond in a positive way over you know what's going on with [our child] and it helps you set aside the sort of small issues that you are having during the day, you know the small stuff suddenly doesn't seem so important. It tends to bring us closer together, I think”*.

Improvements to their “Role as a father” (*n* = 2 utterances), “Relationship with their child” (*n* = 1 utterance) and their “Overall mood” (*n* = 1 utterance) were also conveyed:

*FP3: “[Positive interactions with HCPs] tend to make me act a little more [with my child]. It kind of brings out the best in me as a father.”* *FP3: “Those interactions you do have with HCP, they are very significant, and I think that, I'm sure those professionals they deal with hundreds of people a week, but for us as parents, it's a big deal, so if you have a good interaction and somebody is kind and considerate it definitely can influence your overall mood […].”*

Impacts of negative interactions with HCPs included fathers becoming more “Protective” (*n* = 3 utterances), “Assertive” (*n* = 2 utterances) and “Vigilant” (*n* = 1 utterance):

*FP2: “[Previous negative experiences with HCPs] resulted in the fact that I was checking everything, verifying if there were any errors. When there was even a small error or inconsistency, I would jump on the opportunity to complain and report it. I think this contributed to us receiving the services we needed [for my child].”*

One participant also reported that negative interactions with HCPs had the potential to “Affect subsequent relations with their partner and/or their child” (*n* = 1 utterance):

*FP3: “[…] if you have a negative interaction, it can definitely stick with you and can kind of come out with the wife or the kids.”*

#### Impacts of Fathers in the Healthcare of Their Child With Disability (From the Perspectives of HCPs)

The HCPs reported that the involvement of fathers in the healthcare of their child with a disability had numerous positive impacts (*n* = 6 subthemes, *n* = 30 utterances). The most commonly described effect is that fathers have the potential to “Contribute to the child's development, recovery and well-being” when they are involved and engaged in the healthcare of the child (*n* = 11 utterances):

*HCP05: “I think [fathers] have a significant role […]. We have limited amount of time to facilitate recovery or adaptation with compensatory measures, it only moves the needle so far. If we can extend that with dad's support, so if they are involved if they are supportive, that facilitates I think in general quicker improvement, quicker recovery, quicker restoration of function and independence. And when I've seen it, it's been amazing.”*

Second, the HCPs convey that when fathers are involved, this leads to a “Relief in burden of care for the mother and promotes mother's well-being” (*n* = 9 utterances).

*HCP03: “[Father's contribution to mother's wellbeing] is huge, it's the biggest role that the father can have, because [he is] the immediate partner, and the immediate support system that the mother has, so it would be a huge impact.”*

Several participants mentioned that fathers can promote “Therapy through play and fun” (*n* = 5 utterances) and “Can be very innovative” (*n* = 2 utterances):

*HCP13: “One of my favorite dad moments was working with a child who was not yet a word user. So, I was having dad say the word ‘up' and then toss the kid up in the air, gently, with lots of care and love, and that child did eventually say the word ‘up.' Me and the dad thought that might have been the child's first word after maybe about half an hour of that rough and tumble physical play. This was a nice match of a style of play that the dad was comfortable with. ‘Up' is a great first word because it's easy and meaningful.”* *HCP01: “Dads' ideas are really great and dismissing them or not knowing how they are contributing - I think we are losing a big piece of information. Because you see […] the strategies they think of might be something totally out of your ball game, so they are giving a completely different perspective that could be excellent, so by disregarding them you are missing that.”*

The involvement of fathers can also lead to more “Compliance in therapy” (*n* = 2 utterances) and enhanced “Family dynamics and overall functioning” (*n* = 1 utterance).

*HCP10: “The thought that I had would be the kid who gets really frustrated when either they don't understand what is going on or they can't communicate or both […] if the dad explains to the other kids and to the grandparents ‘here's how we deal with little Johnny,' and if little Johnny feels supported and understood as much as possible, I think that probably has a positive effect on the child's behavior; therefore, the whole family's functioning. It would just sort of reduce the frustration for the child [and will make] everyone a little happier.”*

#### Barriers and Facilitators to Optimal and Empowering Interactions Between Fathers of Children With Disabilities and HCPs

Tables 2A,B outline the emerging subthemes related to barriers and facilitators to optimal and empowering interactions between fathers and HCPs from the perspectives of both study groups. Overall, 147 (*n* = 147) utterances were classified in barriers (*n* = 87 utterances; HCP-reported *n* = 68; father-participants-reported *n* = 19) and facilitators (*n* = 60; HCP-reported *n* = 41; father-participants-reported *n* = 19).

**Table 2A:** Barriers to optimal and empowering interactions and parent–professional relationships between fathers of children with disabilities and HCPs.

**Theme** ***n* = number of utterances** **% of Barriers**	**Subtheme**	**Description**	**Father's utterances** ***n* (% of total)**	**HCPs' utterances** ***n* (% of total)**	**Total utterances** ***n* (% of Theme)**
**Factors related to fathers** ***N* = 34** **39.1%**	Uninvolved or disengaged	Fathers who are uninvolved and/or disengaged in the healthcare of their child (e.g., during medical visits)	0 (0)	7 (100)	7 (20.5)
	Rigid working schedule and stress level	Fathers' working schedule and stress levels that limit the possibility of interactions and involvement in the healthcare of their child.	2 (28.5)	5 (71.4)	7 (20.5)
	Unhelpful personality traits	Fathers' personality traits impeding communication and parent-professional relationship building (e.g., too demanding, dismissive, imposing).	0 (0)	6 (100)	6 (17.6)
	Denial	Fathers who are in denial about their child's challenges or who have difficulty accepting the situation.	1 (16.7)	5 (83.3)	6 (17.6)
	Lack of understanding	Fathers who lack understanding or knowledge about their child and/or about the condition of their child.	1 (25.0)	3 (75.0)	4 (11.7)
	Previous experiences	Fathers with previous negative experiences with HCPs and/or the healthcare system and who are “carrying the baggage” into a new conversation/interaction.	0 (0)	2 (100)	2 (5.8)
	Difficulty opening up	Fathers who have difficulty opening up about certain issues and/or share their feelings and concerns for fear of being perceived “weak.”	0 (0)	1 (100)	1 (2.9)
	Difficulty understanding HCPs	Fathers who have difficulty understanding HCPs for various reasons (e.g., communication barrier, low education level)	0 (0)	1 (100)	1 (2.9)
	**Total**		**4 (11.7)**	**30 (88.2)**	**34 (100)**
**Cultural beliefs** *N* = 18 20.7%	Father's role	The cultural beliefs about father's role in the family and the healthcare of the child.	2 (28.5)	5 (71.4)	7 (38.8)
	HCP who are female	Father's cultural beliefs toward HCPs who are female.	0 (0)	4 (100)	4 (22.2)
	Culture-general	Ethnicity or general cultural factors that can affect interactions.	0 (0)	3 (100)	3 (16.6)
	Disability	Fathers' cultural beliefs about their child's disability.	0 (0)	2 (100)	2 (11.1)
	HCP discipline	Fathers' cultural beliefs about HCP discipline (e.g., physicians vs. therapists).	0 (0)	1 (100)	1 (5.5)
	HCP age/experience level	Fathers' cultural beliefs about HCP of young age and/or possible low level of experience in childhood disability.	0 (0)	1 (100)	1 (5.5)
	**Total**		**2 (11.1)**	**16 (88.8)**	**18 (100)**
**Stereotypical thinking or assumptions** *N* = 9 10.3%	**-**	Refers to preconceived or stereotypical beliefs (e.g., “father is present at the medical visit because of lack of trust or unsatisfaction with services”).	3 (33.3)	6 (66.7)	9 (100)
	**Total**		**3 (33.3)**	**6 (66.7)**	**9 (100)**
**Family dynamics** *N* = 8 9.1%	**-**	Refers to various limiting family dynamics (e.g., overpowering partner).	0 (0)	8 (100)	8 (100)
	**Total**		**0 (0)**	**8 (100)**	**8 (100)**
**Factors related to the healthcare and child-care systems** *N* = 7 8.0%	Accessibility to information, services, or contacts	Challenges related to accessing information, services or contact within the electronic record system/scheduling system.	3 (60.0)	2 (40.0)	5 (71.4)
	Delays in appointments - long waiting times	Long waiting lists for medical appointments.	1 (100)	0 (0)	1 (14.2)
	Maternity vs. paternity leaves	Refers to a longer vs. shorter maternity vs. paternity leave, possibly affecting father's involvement in child's healthcare, general care, knowledge about child and condition.	0 (0)	1 (100)	1 (14.2)
	**Total**		**4 (57.1)**	**3 (42.8)**	**7 (100)**
**Factors related to HCPs** *N* = 6 6.8%	Focus on negative aspects	HCP focusing on negative aspects, disability.	2 (100)	0 (0)	2 (33.3)
	Lack of attention to fathers	HCP not directing attention specifically to fathers in interactions.	1 (50.0)	1 (50.0)	2 (33.3)
	Lack of knowledge about cultural factors and how to bridge them	HCP who lacks awareness about certain cultural factors that can be at play and how to bridge them during interactions.	0 (0)	1 (100)	1 (16.7)
	Early career, confidence level	HCP who is early in their career and have a low confidence level when interacting with fathers.	0 (0)	1 (100)	1 (16.7)
	**Total**		**3 (50.0)**	**3 (50.0)**	**6(100)**
**Time limitation** *N* = 5 5.7%	**-**	Refers to time limitations during interactions (e.g., feeling of being rushed through medical visit or limited appointment time because of ongoing case load of the HCP).	3 (60.0)	2 (40.0)	5 (100)
	**Total**		**3 (60.0)**	**2 (40.0)**	**5 (100)**
	**Grand Total**		**19 (21.8)**	**68 (78.2)**	**87 (100)**

**Table 2B:** Facilitators to optimal and empowering interactions and parent–professional relationships between fathers of children with disabilities and HCPs.

**Theme** ***n* = number of utterances** **% of Facilitators**	**Subtheme**	**Description**	**Father's utterances** ***n* (% of total)**	**HCPs' utterances** ***n* (% of total)**	**Total utterances** ***n* (% of Theme)**
**Factors related to fathers** *N* = 29 48.3%	Engagement	Father who is engaged and involved in child's care and healthcare.	0 (0)	5 (100)	5 (17.2)
	Ability to see the overall picture and practicality	Father who can see the overall picture and be practical with solutions, innovative.	0 (0)	5 (100)	5 (17.2)
	Acceptance	Father who accepts the child's conditions and challenges.	1 (25.0)	3 (75.0)	4 (13.7)
	Knowledge of the child's condition, medical history	Father who had a good understanding and awareness of the child's condition, medical history and child's needs.	0 (0)	4 (100)	4 (13.7)
	Personality	Helpful personality traits (e.g., advocacy, perseverance, authority).	2 (66.7)	1 (33.3)	3 (10.3)
	Responsibilities sharing	Father who is sharing responsibilities with partner (cultural shift in traditionally set roles).	0 (0)	2 (100)	2 (6.8)
	Ability to prioritize	Father who is able to prioritize needs.	0 (0)	2 (100)	2 (6.8)
	Openness to learn	Father who is open to learn.	1 (50.0)	1 (50.0)	2 (6.8)
	Ability share feelings	Father who is able to open up and share feelings, talk about issues.	0 (0)	1 (100)	1 (3.4)
	Working conditions	Employer who understands father's situation and is flexible, allowing father to be involved in child's healthcare needs.	1 (100)	0 (0)	1 (3.4)
	**Total**		**5 (17.2)**	**24 (82.7)**	**29 (100)**
**Factors related to HCPs** *N* = 23 38.3%	Providing explanations	HCP who is providing explanations during interactions with fathers of children with disabilities.	4 (100)	0 (0)	4 (16.6)
	Taking their time in the interactions	HCP who is taking their time during interactions with fathers and do not “rush the appointment.”	1 (25.0)	3 (75.0)	4 (16.6)
	Using family-centered care approach	HCP who is using a family-centered approach, where the needs of all family members are heard and considered, where both parents are treated equally.	3 (75.0)	1 (25.0)	4 (16.6)
	Level of experience (novice vs. seasoned)	HCP with more years of experience in the field of childhood disability.	0 (0)	3 (100)	3 (12.5)
	Having the intent to involve fathers	HCP with intent to involve fathers of children with disability into assessment, treatment plan and/or follow-up.	0 (0)	2 (100)	2 (8.3)
	Reporting improvements, focus on positive aspects	HCP who is not solely focusing on disability and challenges, but who also report on positive aspects and improvements.	1 (50.0)	1 (50.0)	2 (8.3)
	Advocating for family's needs	HCP who is acting as an advocate for the family and the child.	2 (100)	0 (0)	2 (8.3)
	Grabbing the opportunity when father shows interest	HCP who can detect father's interest and take the opportunity to involve them as soon as possible.	0 (0)	1 (100)	1 (4.1)
	Engaging fathers	HCP who is engaging fathers as much as possible.	1 (100)	0 (0)	1 (4.1)
	Having a positive father role model	HCP who themselves have had a positive father role model in their life.	1 (100)	0 (0)	1 (4.1)
	**Total**		**13 (54.2)**	**11 (45.8)**	**24 (100)**
**Communication strategies** *N* = 4 6.6%	Everyday examples	Using examples from everyday life to describe complex situations.	0 (0)	1 (100)	1 (25.0)
	Visual supports	Using visual supports in interactions that require deep understanding and lots of explanation.	0 (0)	1 (100)	1 (25.0)
	Humor	Using humor during interactions, where appropriate.	0 (0)	1 (100)	1 (25.0)
	Concreteness	Emphasizing how a father can help *via* concrete and clear suggestions and explanations why it is helpful.	0 (0)	1 (100)	1 (25.0)
	**Total**		**0 (0)**	**4 (100)**	**4 (100)**
**Long lasting relationship with the same HCP** *N* = 1 1.6%	-	Refers to having a long-lasting parent-professional relationship with the same HCP.	1 (100)	0 (0)	1(100)
	**Total**		**1 (100)**	**0 (0)**	**1 (100)**
**Follow-up** *N* = 1 1.6%	-	Refers to following up with fathers if they are absent from the medical visit in relation to their child's care.	0 (0)	1 (100)	1 (100)
	**Total**		**0 (0)**	**1 (100)**	**1 (100)**
**Accommodating father's schedule** *N* = 1 1.6%	-	Refers to accommodating father's schedule in the attempt to have them present during healthcare visits.	0 (0)	1 (100)	1 (100)
	**Total**		**0 (0)**	**1 (100)**	**1 (100)**
	**Grand Total**		**19 (31.7)**	**41 (68.3)**	**60 (100)**

The most commonly reported barrier to optimal and empowering interactions between fathers and HCPs (*n* = 34 utterances, accounting for 39.1% of all barriers) was found to be associated with *Father-related factors*. Predominantly, accounting for 76.5% of barriers, these factors included fathers who were uninvolved or disengaged; rigid working schedule and high stress level of fathers; certain personality traits (e.g., being demanding, dismissive, or too imposing); and being in denial with regard to condition and challenges of their child:

*HCP01: “There are two general types of dads that are less helpful: one is the one that is uninvolved – that's the one that even if they are present, they might be on their phone the whole time not paying attention. You kind of feel like you are bothering them if you have to ask them a question or if you want to point something out to them that their child is doing…those dads are not that helpful. The second type of dad that is not that helpful is the authoritative dad who thinks that he knows best because he is the parent. I've had a dad pull me out of a room before I see a child and tell me: ‘Tell my kid he can't drive.' whereas that might not be the situation, but he thinks his son cannot drive and therefore I should be conveying the same message. So those are the two general types of dads who are a little less helpful in situations. And also, it puts a strain on the therapeutic relationship because if you aren't going to carry through for ethical reasons or professional reasons you are deemed a bad therapist. And then there is of course stress and work if dads are very-very busy, they may not feel the importance to put down their phone to listen to what we are saying […]. So, they are stressed and thinking of many-many things, they might miss appointments, and you as the health care professional have the impression that they are not involved but it's not necessarily true because they might be very involved at home it's just that they can't miss work. […] Stress and work can really affect the way a dad might come across. Maybe they are stressed about something external, and they are taking it out on whoever is in front of them. You don't know what has happened while they have been waiting in the waiting room.”* *FP06: “Yeah, I feel like my ex-wife was accepting my kid like she was. She was more supportive than I was. I was engaged but I was demanding. She was engaged, but she was supportive. I was trying to push [my child] and my ex-wife was trying to support her. When you come in front of a professional, you can't keep pushing; you have to start accepting so you can help. It took me time. […] I heard quite a bit of that, of people telling me that's not a real issue. So sometimes, the first reaction you get from men is not a very supportive one, so I guess when you go meet a professional if you don't believe in what you are doing and you just doubt the reality of that, it's going to be difficult.”*

The remainder of *Factors related to fathers* included lack of understanding or knowledge of fathers regarding the condition of their child; their previous negative experiences with HCPs and/or the healthcare system; difficulty opening about issues and feelings with the HCP; and difficulty understanding the HCP:

*FP03: “[…] In my experience, [my wife] is always the one with the information and the background knowledge and previous experience with these people and therefore is getting a lot more value out of the meetings. For me, if I don't have the base knowledge of what we are talking about then I'm not going to get as much out of it.”* *HCP09: “So what I find most difficult is when you see that something is bothering [dads] and then they are just not talking. […] It is hard to get them to confide because they do not do that necessarily, that's not in their nature really to share their feelings, so, but you can actually see that something is wrong and they're just not talking about it. So, it's hard to tiptoe around the issue where you know something is not right, but then, that's another thing, a mom would just come out and tell you what's wrong and what's bothering her and then you can talk about it and get it out and try to find solutions, but with dads it's much more difficult to do that.”*

*Cultural beliefs*, such as those about role of a father, disability, and HCP discipline/gender/age-experience level, accounted for 20.7% of all barriers:

*HCP10: “I see a lot more different cultures, and over the years it is often, not always, the mother who is the one who describes. Like the dad sort of drove her there, but he doesn't answer a lot of my questions, often isn't that worried, and sometimes I think that is a cultural thing that it's this is the moms job, the mom raises the kids and I make the money or I'm the provider or am the patriarch, but that's their culture for the father not to be as involved and also not that worried about developmental disabilities.”*

*Stereotypical thinking or assumptions*; *Family dynamics* (e.g., overpowering partner); *Factors related to the healthcare and child-care system* (e.g., wait times, accessibility to services); and *Time limitation* (e.g., feeling rushed through the appointments) were also reported as barriers:

*FP02: “Every time the physiotherapist sees me [the father], she has this face: ‘Oh okay, it's the father…' as if I were there to be mean, to see how you do your job. I have the impression that it looks like when they see the father [at the medical visit], they think it is because there is something wrong.”* *FP03: “One thing that always bothers me is when I sense that there is a rush on the meeting, if there is a rush on the interaction we are having, and if there's not enough thought going into a suggestion. You have just met my kid, you are telling him this thing right here, and it is like hang on, there can be an impact there on me, where I feel like, you know you are more apt to follow someone's advice or hear what they have to say if you believe that they know what you are dealing with, they know your particularities. Going to any HCP where they are trying to rush you out of the office it's not a good situation for any positive interaction.”*

Lastly, certain *Factors related to HCPs* also emerged as obstacles and included: HCPs who are focusing on negative aspects and on disability; those lacking attention to fathers in their interactions; the lack of knowledge about cultural factors and how to bridge them; and being early in their career (e.g., low confidence level in interacting with fathers):

*FP03: “I feel like it's just important to remind [HCPs] that we as parents can really use a dose of positivity, and it feels good when they, I think starting out on a foot of positivity has been great and too often people jump straight into ‘Okay, here's what I'm seeing that's wrong with your son.' […] it's all about what he's sort of failing at, really, and that can be really stressful actually.”* *HCP13: “I've seen professionals that are great at coaching moms […] but in other situations, just totally stone-wall dad. What are you doing? He came to the appointment, like you might hook him!”* *HCP13: “I have a child on my list right now who, they speak [another language]. During one of my feeding visits, dad is very polite and very quiet, just kind of smiles and nods. And in one of my feeding visits, I said ‘I like seeing you, I like seeing you here, I'd love to see you, you know for communication visits, you are so invited, you could come if you wanted to, we'd have fun together,' and then he came to some subsequently which really made [me happy]. But I do not always know how to build that bridge, especially if I do not meet dad in the clinic area in the first place. I don't always know how to build that bridge.”* *HCP10: “When I was new, I was probably intimidated talking to anyone, I felt like an imposter, like I didn't know what I was doing.”*

In terms of facilitators, *Factors related to fathers* were reported 48.3% of the time and included: engagement of father; ability to see the overall picture and practicality; acceptance of the condition of child, strengths and weaknesses; knowledge of the condition of the child; positive personality traits (e.g., advocacy, perseverance); sharing of responsibilities; ability to prioritize; openness to learn; ability to share feelings; and flexible working conditions:

*HCP10: “The [experiences and interactions] that are positive are [with] the involved dads. They are the ones who are asking me questions who seem to know their child quiet well, they are aware of their child's strengths and weakness.”* *HCP11: “So the father would come to some appointments and the positive interaction was [that] he was always [sharing] with me how he felt about the kid. He was always very happy when he came back and he had put into place the recommendations I had given him.”* *HCP01: “[Fathers] still care for their child very-very much, but they aren't caught up in their insecurities, and their guilt, and their anxieties. They are able to take a step back and really see objectivity what is important in this moment for their child.”* *HCP12: “…Father's openness and willingness to receive information will have a play on how comfortable I am too. So, with the fathers who are really interested and want to know why we are doing certain activities, [the interaction] is really not a problem.”*

Several *Factors related to HCPs* emerged as enablers and included: providing explanations; taking their time in the interactions; using a family-centered approach; level of experience; intent to involve fathers; reporting improvements and focus on positive aspects; advocating for needs of the family; grabbing on the opportunity when the father shows interest; engaging fathers; and having a positive father role model in their lives. Seventy percent (70%) of all the utterances of the father-participants in the theme “Facilitators” were found to be in this category (i.e., *Factors related to HCPs*):

*FP04: “I would more say was the one where we did the occupational therapy where the HCP was actively engaged with the parents, they showed us the techniques to do at home in order to help Jackson when he is at home not just at the therapy sessions, so for me they were engaged with us.”* *FP03: “[One facilitator to optimal interactions is to] try as best as you can to give time to the interaction, to provide knowledge in the meeting itself for when it's needed, like if, I mean it's definitely not on them to educate us every time, but it's helpful and I appreciate it.”* *FP02: “[In the best interactions], we [father and mother] were considered both as parents of equal value, and not ‘the mother and the father who is on the side.”'*

Further, *Communication strategies* were reported as positive (100% by HCPs) and included: the use of everyday examples; visual supports; and humor; and concreteness, where the HCP can emphasize how a father can help using clear suggestions and explanations as to why it is beneficial.

*HCP09: “So a lot of the good interaction that I find is when we do teach or we're trying to solve issues, they are very engaged in that. So, to give them concrete explanations, to give them concrete things to do, like, that's what they like, and that's what they're engaged into.”* *HCP07: “I use humor a lot with fathers, they get on well. Sometimes when we use a little bit with humor, it creates good interactions.”*

The remaining facilitators included having a *Long-lasting relationship with the same HCP*; *Following-up with fathers* when they are absent from the medical visit; and *Accommodating father's schedule* in attempt to have them present during healthcare visits.

#### Solutions to Optimize Interactions Between Fathers of Children With Disabilities and HCPs and Involvement of Fathers in the Healthcare of Their Child

Seventy (*n* = 70) utterances (*n* = 18, 25.7% from the father-participants; *n* = 52, 74.3% from the HCPs) were categorized into the theme of *Solutions to optimize interactions between fathers of children with disabilities and HCPs and overall father's involvement in the healthcare of their child*. Table 3 outlines the reported solutions. Most solutions (65.7%) were proposed directly for HCPs and included: *Changes to communication strategies*; *assessment, treatment and follow-up*; *Appointments and scheduling*; *Fair consideration of both parent figures*; and *consideration of cultural differences and values*.

**Table 3:** Solutions to improve interactions and parent–professional relationships between fathers of children with disabilities and HCPs.

**Theme** ***n* = number of utterances** **% of Solutions**	**Subtheme *n* = number of utterances % of Theme**	**Sub-subtheme**	**Description**	**Father's** **utterances** ***n* (% of total)**	**HCPs' utterances** ***n* (% of total)**	**Total utterances** ***n* (% of Theme)**
**Solutions for HCPs** *N* = 46 65.7%	*Communication strategies* *N* = 20 43.5%	Inclusion and direct attention	To include fathers in conversations and direct attention to them in interactions.	1 (12.5)	7 (84.5)	8 (40.0)
		Time	To take more time when interacting with fathers.	1 (33.3)	2 (66.7)	3 (15.0)
		Adjustment, individualization	To adjust communication strategies to father's educational level, culture, values, comprehension level, and interests.	1 (33.3)	2 (66.7)	3 (15.0)
		Positive focus	To focus on strengths and positive aspects.	2 (100)	0 (0)	2 (10.0)
		Practicality	To make conversation practical for fathers and outline how can they help, be educational.	1 (50.0)	1 (50.0)	2 (10.0)
		Active listening	To listen actively to fathers and their observations.	0 (0)	1 (100)	1 (5.0)
		Clear expectations	To outline expectations from the start.	0 (0)	1 (100)	1 (5.0)
	**Total**			**6 (30.0)**	**14 (70.0)**	**20 (100)**
	*Assessment, treatment and follow-up* *N* = 12 26.1%	Involvement in intervention session	To involve fathers in interventions sessions.	1 (16.7)	5 (83.3)	6 (50.0)
		Involvement in assessment sessions.	To involve fathers in assessments and screening sessions.	0 (0)	2 (100)	2 (16.6)
		Visual supports for home programs.	To develop visual supports for home programs and include pictures of male figures.	2 (100)	0 (0)	2 (16.6)
		Follow-up	To follow up with fathers if they are absent from the session by providing updates and keeping them informed.	0 (0)	1 (100)	1 (8.3)
		Assessment setting modification	To modify assessment setting by including a double-sided mirror.	0 (0)	1 (100)	1 (8.3)
	**Total**			**3 (25.0)**	**9 (75.0)**	**12 (100)**
	*Appointments and scheduling* *N* = 9 19.5%	Agenda	To consider both parents' agendas and accommodate father's schedule for him to be present.	1 (14.3)	6 (85.7)	7 (77.7)
		Crucial timepoints	To insist on seeing both parents at crucial timepoints (initial assessment, diagnosis, planning for treatment, re-assessments).	0 (0)	2 (100)	2 (22.2)
	**Total**			**1 (11.1)**	**8 (88.8)**	**9 (100)**
	*Fair consideration of fathers vs. mothers* *N* = 4 8.6%	-	HCP to offer fair attention to fathers and mothers by considering them as equal parent-partners.	1 (25.0)	3 (75.0)	4 (100)
	**Total**			**1 (25.0)**	**3 (75.0)**	**4 (100)**
	*Consideration of cultural differences and values* *N* = 1 2.1%	-	HCP to consider cultural difference and values that might be at play (their own vs. patient's) and be sensitive to those factors when interacting with families.	0 (0)	1 (100)	1 (100)
	**Total**			**0 (0)**	**1 (100)**	**1 (100)**
**Education and awareness** *N* = 11 15.7%	*Formal education curriculum for trainees* *N* = 6 54.5%	-	Refers to the development and inclusion of an educational module (for trainees) that is specific to family-centered care in the formal curriculum within the health-sciences programs (pediatric-related sections).	0 (0)	6 (100)	6 (54.5)
	*Continuing education for HCPs* *N* = 4 36.4%	-	Refers to the development and launch of a continuing educational module that is specific to family-centered care for practicing clinicians in the field of childhood disability.	0 (0)	4 (100)	4 (36.3)
	*Fathers' awareness* *N* = 1 9.0%	-	Refers to the development and launch of knowledge translation material to raise awareness of fathers (and other family members) about their positive contributions to child's health and family's well-being when they are actively involved and engaged in child's healthcare and upbringing.	0 (0)	1 (100)	1 (9.1)
	**Total**			**0 (0)**	**11 (100)**	**11 (100)**
**Healthcare services and media** *N* = 11 15.7%	*Access* *N* = 5 45.4%	-	Refers to improving access, transparency and visibility of information and services.	5 (100)	0 (0)	5 (45.4)
	*Public relations (media, titles of groups/programs)* *N* = 4 36.6%	-	Refers to changing how fathers are portrayed in the media and in materials used by the healthcare settings and modify the titles of groups/program to be inclusive of fathers.	0 (0)	4 (100)	4 (36.3)
	*Medical records/administration* *N* = 2 18.2%	-	Refers to minimizing administrative and medical record biases (e.g., primary phone number is always that of the mother) to accommodate both parents and promote inclusiveness.	1 (50.0)	1 (50.0)	2 (18.1)
	**Total**			**6 (54.5)**	**5 (45.5)**	**11 (100)**
**Solutions for fathers** *N* = 2 2.8%	*Engagement* *N* = 1 50%	-	Refers to fathers to actively engage in conversations during interactions with HCPs.	1 (100)	0 (0)	1 (50.0)
	*Awareness* *N* = 1 50%	-	Refers to fathers gaining awareness about their potential impacts of involvement in child's healthcare.	0 (0)	1 (100)	1 (50.0)
	**Total**			**1 (50.0)**	**1 (50.0)**	**2 (100)**
	**Grand Total**			**18 (25.7)**	**52 (74.3)**	**70 (100)**

For *Changes to communication strategies*, the participants recommended: including fathers in conversations and directing attention to them; taking time in conversation and avoid rushing patients; adjusting communication strategies to the educational level of fathers, culture, values, comprehension level and interest; focusing on positive aspects; being practical and outlining how fathers can help; listening actively to observations of fathers; and clearly outlining expectations from the start.

Several suggestions on how the process of *Assessment, treatment*, and *follow-up* can be improved emerged and included: involving fathers in the intervention and assessment sessions as much as possible, providing visual supports in home programs, following-up with fathers if they are absent, and modifying the assessment setting (by including a double-sided mirror) to alleviate the stress related to the presence of HCP during an assessment conducted *via* observation (e.g., playtime, parent-child interaction, feeding). *Considering both parents' agendas* and *Accommodating fathers' schedules* to promote their presence at medical visits was also reported as one solution. The HCPs also suggested the need to be *Sensitive to cultural differences* and values during interactions and adjust their approach accordingly.

Solutions related to *Education and awareness* were reported by the HCPs only (100%) and included developing and launching: (1) formal education curriculum for trainees with a focus on family-centered care and particularities related to fathers; (2) continuing education material for practicing HCPs in the field of childhood disability, outlining current issues and how to bridge them; and (3) material targeting fathers of children with disability, with an aim to increase their awareness about potential positive outcomes of their involvement and engagement in the healthcare and upbringing of their child.

Changes to *Healthcare services* at large (e.g., improving access, transparency, and visibility of information; medical records, and administration) were proposed entirely by the father-participants (100%). In addition, modifications to *how fathers are portrayed* in the media and in the healthcare system (e.g., program titles) were recommended:

*HCP13: “So I think also that when we see children represented in the media, they are with mums, and then when we see children with disabilities represented in the media they are with mums and then like if we even manage to get a representation of a dad, we don't often see pictures and videos of dads with kids with disabilities. And if we do, we do not see a spectrum of dad abilities – we get hero- dad. We do not see ordinary dads doing the ordinary things that come with kids with disabilities. I think representation matters and representation of dads who maybe are not superheroes, maybe they are just regular dads who play in regular ways. When dads exist in the media with kids, they are either amazing at that play or they are described as amazing even when they are just doing their jobs. I think we hit both sides of that so I think dads can either get intimidated, like if I am not YouTube worthy, I best not jump in. Or yeah just they don't know that people that look like them can be a part of the picture.”* *HCP12: “[What was helpful is] for example, our toddler group is called ‘Moms and Tots' and recently we changed it to ‘Parents and Tots.' I find it makes a difference, the fathers that started to get included they kept asking: Why it is called ‘Moms and Tots,' and so little things like that…”*

Lastly, solutions *Targeting fathers* emerged and included: increasing engagement and awareness of fathers about their potential impacts of engagement in the healthcare of their child.

## Discussion

To enhance healthcare-related interactions and experiences for fathers of children with developmental disabilities, the goal was to explore the barriers to and facilitators of positive and empowering interactions and parent–professional relationships, from the perspectives of fathers of children with developmental disabilities and HCPs. The participants of this study reported numerous impacts of positive interactions and involvement of fathers in the healthcare of their children. Also, we determined several influential factors (obstacles vs. enablers) that are at play during parent–professional interactions. We also outlined practical solutions that can be implemented in various clinical settings with the aim of improving interactions and parent–professional relationships, as well as promoting family-centered care.

In this study, the fathers reported to be moderate to very much involved in the healthcare of their child (e.g., arranging healthcare services and being present during medical visits) and other activities (e.g., home program, feeding, exercises). This is congruent with results from previous qualitative studies on fathering children with disabilities (19–21). Nonetheless, the HCP-participants conveyed that fathers are only somewhat involved in those activities. The discrepancy in findings between the two study groups could be attributed to selection and response biases. To explain, the interviewed HCPs have experience with numerous fathers of children with disabilities with different engagement levels (involved vs. not involved). On the other hand, the fathers who participated voluntarily in this study are parents who are moderate to very much engaged in the healthcare and upbringing of their child. However, previous research has suggested that, resulting from cultural beliefs about parenting roles in the family, some fathers of children with disabilities may choose to be less involved with their children. It is common for the mother to undertake the role of primary caregiver and the care from the father related to the child becomes optional (22). Similarly, we also found that cultural factors, such as the traditional beliefs about the role of the father in the upbringing and healthcare of the child, represented 20.7% of all reported barriers and were predominantly conveyed by the HCPs (88.8% of the time). Furthermore, the fathers reported that although they are overall satisfied with the interactions they have had with HCPs in the past, they report that HCPs are only sometimes attentive to them during interactions. This finding resonates with previous research where many fathers report feeling “invisible” during interactions with service providers in relation to the care of their child (23, 24).

Personal factors related to fathers (e.g., their level of engagement/involvement, personality traits, denial vs. acceptance, difficulty opening up about issues) accounted for most of the obstacles or enablers to optimal interactions and parent–professional relationships. Those factors may originate from and be further exacerbated by the high stress levels and mental health issues potentially experienced by fathers of children with disabilities. For example, Giallo et al. (25) demonstrated that in fathers of children with disabilities, there were high rates of symptoms of depression and stress, with nearly 8% reporting severe to extreme symptoms. A recent Canadian study using population-level administrative data from the Ministry of Health compared the mental health of parents of children who have a DD with the mental health of parents of typically developing children. They reported that diagnosed mental health issues were the most prevalent health issues among parents of children with disabilities (including fathers at 60%) (26). The findings of this study regarding external factors, such as supportive vs. hindering work conditions, resonate with previous research suggesting that parents of children with disability experience different stressors, capacity to work, and patterns of pastimes compared with parents of typically developing children (27). It is, therefore, important for HCPs to be aware of those elements and to be sensitive to potential mental health challenges and lived stressors when interacting with fathers of children with disabilities.

Further, the father's denial vs. acceptance of the child's condition, strengths, and challenges was identified as an important influential factor by both study groups. Comparatively, a recent study explored experiences of fathering children with autism spectrum disorder (19). In this study, fathers described their experiences as a “path toward acceptance.” Hence, we propose that HCPs support fathers in their journey toward acceptance and this might lead to enhanced connections and experiences.

Similar to previous findings, where fathers report feeling overwhelmed and overshadowed during interactions with HCPs (22, 24), we found that factors, such as family dynamics (e.g., overpowering partner), factors related to HCPs (e.g., focusing on negative aspects, lack of knowledge about cultural factors, lack of attention to fathers), and time limitation, where fathers feel rushed through the medical visits, are hindering interactions and parent–professional relationships. The study participants also reported several issues with the healthcare system in general (e.g., long waiting times, lack of visibility of services and transparency), and that previous negative experiences with the healthcare system shaped them into being more vigilant, defensive, or protective. Analogous results have been reported in the past, where fathers of children with disabilities saw themselves as “advocates fighting obstructive services to access appropriate care” (19). On the other hand, when HCPs provide explanations, take their time during interactions, implement family-centered approaches in care, demonstrate intent to involve fathers, advocate for the family, and engage fathers whenever possible, it enables interactions and builds and maintains parent–professional relationships.

Finally, in line with identified barriers and facilitators, several practical solutions emerged. These solutions can be easily implemented in the clinical settings of today to enhance the experiences of fathers of children with disabilities and promote helpful and empowering interactions between fathers and HCPs. The majority of the suggestions pertain to communication strategies (e.g., directly including fathers and paying attention to fathers in sessions), changes in clinical practices related to evaluation, treatment, administrative system (scheduling), family-centered care (where both parents are considered equally), and consideration of cultural differences. Access to information and services, as well as how fathers are portrayed vs. not portrayed in the media and within the healthcare system should also be evaluated and adjusted as needed. We propose that future research should focus on promoting the development and launch of knowledge translation tools to include educational materials to increase knowledge and awareness of trainees, practicing clinicians, and parents about fathers of children with disabilities.

There are limitations in this study, such as the small sample size of father-participants, potentially limiting the generalizability of the findings. Nonetheless, the data analysis showed saturation of ideas with the included participants. To the advantage of the authors, the sample also included fathers of children from a wide age range and variability in diagnoses, single and married individuals, and of different educational backgrounds. The use of surveys and interviews enabled us to triangulate perspectives through mixed methods, allowing a richer understanding of the experiences of fathers and HCPs. In addition, given the use of patient-oriented research strategy, the study greatly benefited from the inclusion of two father-advisors on the research team, boosting the relevance and suitability of measurement strategies and interpretation of findings.

## Data Availability Statement

The raw data supporting the conclusions of this article will be made available by the authors upon request, without undue reservation.

## Ethics Statement

The studies involving human participants were reviewed and approved by The Research Institute of the McGill University Health Center and University of British Columbia. The participants provided their written informed consent to participate in this study.

## Author Contributions

TO drafted the first version of the manuscript. All the authors developed the study protocol, reviewed the manuscript, provided modifications, and suggestions where appropriate.

## Conflict of Interest

The authors declare that the research was conducted in the absence of any commercial or financial relationships that could be construed as a potential conflict of interest.
