# Supplementary material for: Fathers Matter: Enhancing Healthcare Experiences Among Fathers of Children With Developmental Disabilities
Source: Front Rehabil Sci. 2021 Jul 21;2:709262. doi: 10.3389/fresc.2021.709262 (PMC9397890; doi:10.3389/fresc.2021.709262)
Supplement: Supplementary file 1 [file Data_Sheet_1.PDF]

CHILD-BRIGHT Network

Centre universitaire de santé McGill McGill University Health Centre

Interview package: HEALTH CARE PROVIDERS

Dear participant, please take a moment to fill out the following information about you and your work experience. We are collecting this information for descriptive purposes, as well as to better understand your responses. Providing this information is voluntary.

**Age (years)**	
**Gender**	☐Male ☐Female
**Occupation and status**	☐Full-time ☐Part-time ☐Unemployed ☐Retired ☐On disability
**Highest degree obtained & year of graduation**	
**Work description**	**Population served and setting**:<br>**Experience in childhood disability (yy/mm):**<br>**Time spent on professional development (hrs/month):**

We are interested in hearing about your participation and your experiences of interacting and communicating with fathers of children with disabilities when they accompany their child to and from health-care services that you are providing. For each statement, please select the description that fits you best. Space under each statement is made available to you for other information you want us to know (optional).

	**Not at all- Never**	**Slightly- Sometimes**	**Moderately- Often**	**Very much- All the time**
The following statements are about: Your satisfaction with the interactions/communications you have with fathers and how you think they perceive it when they accompany their child to receive the health-services that you are providing.				
**Overall, I am satisfied with the interactions/communication I had with fathers of children with disabilities in the past.**	☐	☐	☐	☐
**Fathers of children with disabilities are generally attentive with me during interactions.**	☐	☐	☐	☐
**I feel comfortable interacting with fathers of children with disabilities.**	☐	☐	☐	☐
**I feel that fathers of children with disability understand me.**	☐	☐	☐	☐
**I feel that fathers of children with disabilities feel supported by me.**	☐	☐	☐	☐
**I feel that I form/continue a good relationship with fathers of children with disabilities.**	☐	☐	☐	☐

The following statements are about: your perceptions of fathers' involvement, capabilities and advocacy skills.<br>**The extent to which fathers of children with disabilities:**				
	**Not at all- Never**	**Slightly- Sometimes**	**Moderately- Often**	**Very much- All the time**
**…. advocate for their child.**	☐	☐	☐	☐
**… implement and apply your recommendations/treatment regiments you are providing them with.**	☐	☐	☐	☐
**…. are involved in setting up/arranging/organizing health-care services for their child.**	☐	☐	☐	☐
**…. are involved in taking their child to his/her health-care services appointment(s) or emergency visit(s).**	☐	☐	☐	☐
**…. are present when their child is receiving health-care services (alone or with spouse/partner).**	☐	☐	☐	☐

HCP ID number	
Province of professional practice	☐QC ☐BC
Date of interview (dd/mm/yy):	
Interview start time:	
Interview end time:	
Duration of interview:	

During the audio-recorded interview, the interviewer will be asking you the following questions. This is just a guide for the interviewer, and additional questions might be asked for clarification. You can take time before the interview to think about your answers. Text boxes are provided as an option to draft your responses prior to the interview.

1) **In your practice, how involved are you with fathers of children with disability?**

2) **Can you describe, in general terms, your experiences of interacting with fathers of children with disabilities?**

**What types of interactions have been helpful (positive/good experiences) and why?**	**What type of interactions have been less helpful/not helpful at all and why?**

3) How do you believe fathers might contribute to the wellbeing of…:

…their child?	…their child's mother?	…their family as a whole?

4) What conditions do you think might make fathers' inclusion and involvement better? What are some obstacles or challenges to their involvement?

5) What would you have to do/need to have to make space for fathers in your practice?

6) What type of information or support would you need to make space for fathers in your practice?

7) **What does your institution need to do to support inclusion of fathers in your practice?**

8) **Summary/closing remarks/check for saturation of ideas**.
