# Supplementary material for: Fathers Matter: Enhancing Healthcare Experiences Among Fathers of Children With Developmental Disabilities
Source: Front Rehabil Sci. 2021 Jul 21;2:709262. doi: 10.3389/fresc.2021.709262 (PMC9397890; doi:10.3389/fresc.2021.709262)
Supplement: Supplementary file 2 [file Data_Sheet_2.PDF]

FATHERS MATTER Research Project

Interview package for participating fathers

Dear participant, please take a moment to fill out the following information about your and your family. We are collecting this information for descriptive purposes, as well as to better understand your responses. Providing this information is voluntary.

**Age**	**years old**
**Race/ethnicity**	☐Caucasian ☐Black/African ☐Asian ☐Native ☐Hispanic/Latino<br>☐Other:
**Relation to the child**	☐Biological ☐Foster ☐Step ☐Male legal guardian
**Marital status**<br>**more than one option might apply	☐Married ☐Single ☐Divorced ☐Blended family (e.g. remarried)<br>☐Widowed ☐Same-sex couple
**Education level**	
**Employment: occupation & status**	**Occupation**:<br>☐Full-time ☐Part-time ☐Unemployed ☐Retired ☐On disability
**Child(ren) history**	**Number of children**: **Number of children with disability**:<br>**Age rank of child(ren) with disability (e.g. 1st, 2nd, 3rd child)**:<br>**Age of child(ren) with disability**:<br>**Condition (if known) of child(ren) with disability**:<br>**Time of diagnosis (mm/year)**:

We are interested in hearing about your participation and your experiences of interacting and communicating with health-care providers (HCPs) when accompanying your child to and from health-care services. HCPs are certified professionals working in the field of health-care and include: physicians (e.g. pediatrician, neurologist, psychiatrist), rehabilitation specialists (speech language pathologist, occupational/physical therapists), psychologists, special educators, nutritionists, nurses, social workers, etc. For each statement, please select the description that fits you best. Space under each statement is made available to you for other information you want us to know (optional).

	**Not at all- Never**	**Slightly- Sometimes**	**Moderately- Often**	**Very much- All the time**
The following statements are about your involvement in: arranging and accompanying your child to and from health-care services; taking part in your child's recommended therapies; and advocacy practices for your child.				
**I am involved in setting up/arranging/organizing health-care services for my child.**	☐	☐	☐	☐
**I am involved in taking my child to his/her health-care services appointment(s) and/or emergency visit(s).**	☐	☐	☐	☐
**I am present (with my spouse/partner, if applicable) when my child is receiving health-care services.**	☐	☐	☐	☐
**I advocate for my child's health-services** (e.g. I am asking for/about health services for my child, I am reaching out to health professionals about my child, I am following-up with health professionals about my child).	☐	☐	☐	☐
**I am taking part in applying treatment recommendations that my child receives** (e.g. giving medication, providing play/respiratory/stretching/speech/behavioral therapy exercises).	☐	☐	☐	☐

The following statements are about your satisfaction with the interactions/communications you have with the HCPs when accompanying your child to and from health-care services.				
	**Not at all-Never**	**Slightly-Sometimes**	**Moderately-Often**	**Very much-All the time**
**Overall, I am satisfied with the interactions/communication I had with HCPs in the past**.	☐	☐	☐	☐
**HCPs are generally attentive to me.**	☐	☐	☐	☐
**I feel generally comfortable with the HCPs.**	☐	☐	☐	☐
**I feel generally understood by the HCPs.**	☐	☐	☐	☐
**I feel generally supported by HCPs.**	☐	☐	☐	☐
**I feel that generally I formed and continue to have good relationships with the HCPs.**	☐	☐	☐	☐
**I had positive/good experiences/helpful interactions with HCPs.**	☐	☐	☐	☐
**I had negative/bad experiences/not helpful interactions with HCPs**	☐	☐	☐	☐

Father study ID number	
Province of residence	☐QC ☐BC
Date of interview (dd/mm/yy):	
Interview start time:	
Interview end time:	
Duration of interview:	

Sample interview questions

During the audio-recorded interview, the interviewer will be asking you the following questions. This is just a guide for the interviewer, and additional questions might be asked for clarification. You can take time before the interview to think about your answers. Text boxes are provided as an option to draft your responses.

1) **Can you describe, in general terms, your experiences of interacting with HCPs:**
   a. **What types of interactions have been helpful (positive experiences) and why?**

   b. **What types of interactions have been less helpful or not helpful at all (negative/bad experiences) and why?**

2) **How do you think these interactions influence your daily life?**

**Helpful/positive experiences**	**Less helpful/not helpful at all/bad experiences**

3) **How do these interactions influence how you communicate or relate with other HCPs?**

**Helpful/positive experiences**	**Less helpful/not helpful at all/bad experiences**

4) **How do those experiences influence your interactions with your child?**

Helpful/positive experiences	Less helpful/not helpful at all/bad experiences

5) **How those experiences influence your interactions and your relationships with other family members?**

Helpful/positive experiences	Less helpful/not helpful at all/bad experiences

6) **Based on your experiences, and the experiences of other fathers you might know, what types of obstacles or difficulties do you think fathers face in relation to their experiences with their child's HCPs?** Probes: work, pediatric healthcare environment, relationships.

7) **Based on your experiences, and the experiences of other fathers you might know, what facilitates fathers' experiences in communicating interactions with their child's HCPs?** Probes: knowledge about child's

development/condition/health-care services, environment, technology, present members during communications, frequency/duration of contact, HCPs' characteristics.

8) **Based on your experiences, and the experiences of other fathers you might know, what would you like to tell health care providers about working with fathers of children with disabilities?**

9) **Based on your experiences, and the experiences of other fathers you might know, what should people involved in developing programs and services for families know about working with fathers of children with disabilities?**

10) **Summary/closing remarks/check for saturation of ideas**.
